# Crystal structures of the bifunctional tRNA methyltransferase Trm5a

**DOI:** 10.1038/srep33553

**Published:** 2016-09-15

**Authors:** Caiyan Wang, Qian Jia, Ran Chen, Yuming Wei, Juntao Li, Jie Ma, Wei Xie

**Affiliations:** 1 State Key Laboratory for Biocontrol, School of Life Sciences, The Sun Yat-Sen University, Guangzhou, Guangdong 510275, People’s Republic of China; 2 Center for Cellular & Structural biology, The Sun Yat-Sen University, 132 E. Circle Rd., University City, Guangzhou, Guangdong 510006, People’s Republic of China; 3 State Key Laboratory of Optoelectronic Materials & Technologies, School of Physics, Sun Yat-Sen University, Guangzhou, Guangdong 510006, China

## Abstract

tRNA methyltransferase Trm5 catalyses the transfer of a methyl group from S-adenosyl-L-methionine to G37 in eukaryotes and archaea. The N1-methylated guanosine is the product of the initial step of the wyosine hypermodification, which is essential for the maintenance of the reading frame during translation. As a unique member of this enzyme family, Trm5a from *Pyrococcus abyssi* (PaTrm5a) catalyses not only the methylation of N1, but also the further methylation of C7 on 4-demethylwyosine at position 37 to produce isowyosine, but the mechanism for the double methylation is poorly understood. Here we report four crystal structures of PaTrm5a ranging from 1.7- to 2.3-Å, in the apo form or in complex with various SAM analogues. These structures reveal that Asp243 specifically recognises the base moiety of SAM at the active site. Interestingly, the protein in our structures all displays an extended conformation, quite different from the well-folded conformation of Trm5b from *Methanocaldococcus jannaschii* reported previously, despite their similar overall architectures. To rule out the possibilities of crystallisation artefacts, we conducted the fluorescence resonance energy transfer (FRET) experiments. The FRET data suggested that PaTrm5a adopts a naturally extended conformation in solution, and therefore the open conformation is a genuine state of PaTrm5a.

Diverse modifications in tRNAs serve various purposes including promoting translational fidelity^1^,^2^,^3^. Of over 100 identified modifications to date ranging from simple methylations to hypermodifications, the modifications present in the anticodon loop are the most complex, and their biosyntheses usually require the participation of multiple enzymes^4^,^5^. One group of such complex modifications is the wyosine derivatives composed of the tricyclic imidazo-purine that are found exclusively at position 37, 3′ adjacent to the anticodon of tRNA^Phe^ in eukaryotes and archaea^6^. In archaea, G37 hypermodification in tRNA^Phe^ leads to wyosine derivatives (Fig. 1). They are important in reading-frame maintenance during protein synthesis, while the absence of such modifications results in elevated error rates in +1 frame-shifting^7^,^8^. Among the modification products, 7-methylwyosine (mimG) is perhaps the earliest and minimalist version of the wyosine derivatives unique to some archaea, and 4-demethylwyosine (imG-14), isowyosine (imG2) have also been identified as intermediates along the pathway^9^,^10^ (Fig. 1). The first biosynthetic step of mimG is the formation of m^1^G37, catalysed by the S-adenosine-L-methionine (SAM)-dependent tRNA methyltransferase named Trm5^11^,^12^, which belongs to class-I methyltransferases^13^,^14^. The second step is the complex radical-mediated formation of imG-14, catalyzed by the radical SAM enzyme Taw1. Interestingly, Crécy-Lagard *et al.* recently discovered that the Trm5 enzyme from the archaea *Pyrococcus abyssi* (PaTrm5a) also catalyzes the methylation of C7 on imG-14 to produce imG2^11^, which is further methylated on the N4 position of the imidazo-purine ring by Taw3 to form mimG.

During the evolutionary process, some euryarchaeota like *Thermococcus* and *Pyrococcus* preserved both the *trm5* genes from the crenarchaeal origin as well as the native copy, but others apparently lost the latter. Phylogenetic distribution analyses of *trm5* homologues in archaeal genomes allow the identification of three archaeal Trm5 (aTrm5) subfamilies: Trm5a, Trm5b, and Trm5c^11^. Trm5b refers to the native form, while Trm5a refers to the crenarchaeal origin, and Trm5c to other members with divergent Trm5 sequences^11^. The three Trm5s differ substantially in primary sequences. In addition, about 90 residues that exist in all known Trm5bs and Trm5cs at their N-termini are absent from some Trm5as^11^. aTrm5s have the Rossmann fold at the C-termini for catalysis, with the consensus NPPY motif located in the fourth β-strand of the Rossmann fold. The NPPY motif is known to be important in positioning the nitrogen atom of G37 in Trm5, but variations on the consensus sequence have also been discovered^15^,^16^.

The crystal structures of Trm5b from *Methanocaldococcus jannaschii* (MjTrm5b) in complex with the methyl donor analogue sinefungin and adenosine were determined at 2.2-Å and 3.05-Å respectively (PDBs 2YX1 and 3AY0)^17^,^18^. MjTrm5b is composed of three structural domains named D1, D2, and D3 respectively. D3 displays the hallmark Rossmann fold, and makes contacts with both D1 and D2. D2 is the Trm5-defining domain, and exhibits structural similarity to some class-I methyltransferases^13^,^14^. In contrast, D1 has no structural resemblance to any other methyltransferases, and is connected to D2 by a linker that is mostly disordered. Following this work, the crystal structures of MjTrm5b complexed with the substrates tRNA^Leu^ and tRNA^Cys^ were also solved (PDBs 2ZZM and 2ZZN respectively). In both structures, D1 acts like a clamp and contacts the elbow region of tRNA, while D2 recognises G37 and the anticodon loop. D3 stabilises SAM during its binding and catalyses the methyltransfer reaction. The recognition of the SAM at the active site is achieved by hydrogen bonding interactions with Asp223, Asp251 and Val252, and is further reinforced by the stacking interactions with Phe203 and Ile224^19^.

The archaea *Pyrococcus abyssi* carries two Trm5 copies: Trm5a (PAB_RS00630) and Trm5b (PAB_RS03940). Trm5a from *Pyrococcus abyssi* (PaTrm5a) is a newly discovered methyltransferase that catalyses two distinct reactions in tRNA: the N1-methylation of G and the C7-methylation of imG-14 at position 37^11^ (Fig. 1). The formation of the respective products m^1^G and imG2 has been demonstrated by *in-vitro* activity assays using G- or imG-14-tRNA^Phe^ as the substrates^12^. In contrast, under identical experimental conditions, the methylation activity on imG-14 has not been detected with PaTrm5b^11^. The structural basis of the bifunctional substrate specificity of PaTrm5a is interesting but currently unclear. In this study, we solved the crystal structures of apo-PaTrm5a and PaTrm5a in complex with 5′-methylthioadenosine (MTA), S-adenosyl-L-homocysteine (SAH), and adenosine (ADN) respectively (the chemical structures of these ligands were shown in Supplementary Fig. S1). In our crystal structures, D3 displays the typical Rossmann fold. However, the free N-terminal domain D1 is structurally independent of D3, in stark contrast with the D1 conformation in the MjTrm5b structure^17^. The naturally extended conformation exhibited by PaTrm5a was confirmed by the fluorescence resonance energy transfer (FRET) experimental data.

## Results

### Overall structure of apo-PaTrm5a

**Figure 2:**
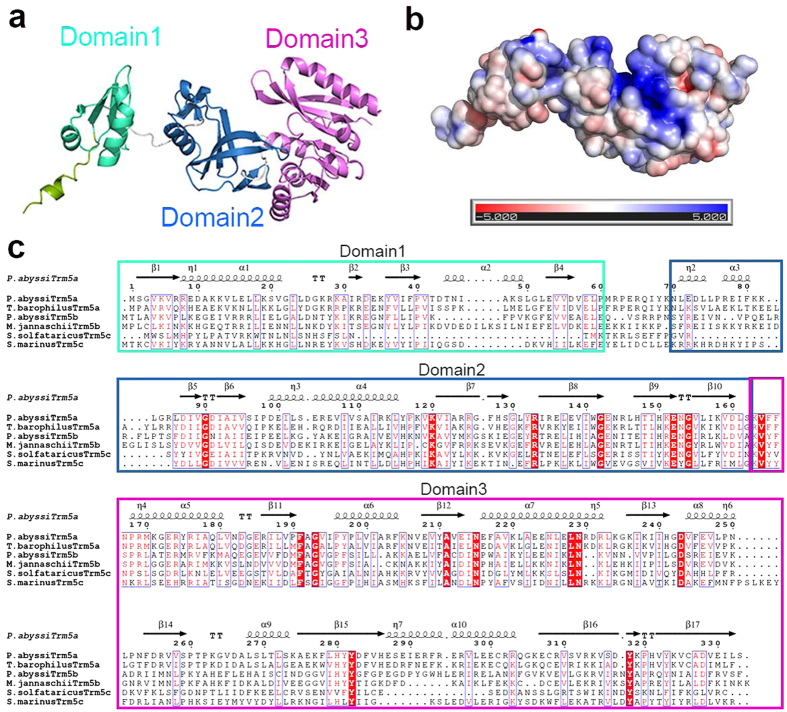
The overall structure of the PaTrm5a and the multiple sequence alignment of the representatives from three archaeal Trm5 subfamilies. (**a**) A ribbon diagram that shows the overall structure of apo-PaTrm5a. The three domains D1–D3 are colored green, blue, and magenta respectively. (**b**) The apo structure of PaTrm5a in the surface-charge rendition, calculated by APBS. (**c**) A multiple sequence alignment of archaeal Trm5s from several archaeal model organisms. Abbreviations used: P. abyssi, *Pyrococcus abyssi*; T. barophilus, *Thermococcus barophilus*; M. jannaschii, *Methanocaldococcus jannaschii*; S. solfataricus, *Sulfolobus solfataricus* and S. marinus, *Staphylother musmarinus*. The secondary structure elements of PaTrm5a are labeled above the sequences. The domains are in colored boxes as described in Fig. 2a.

We successfully obtained four types of crystals of PaTrm5a, including the apo form as well as the complexes with three different ligands. We did not remove the his6-tag from the N--termini, and the crystals diffracted to relatively high resolutions. In all crystals structures, the asymmetric unit contains only one protein molecule. In apo-PaTrm5a, all 333 amino acids were observed in the electron density map, and the uncut tag sequence HHHHHHLEVLFQGPH preceding the target protein was also clearly visible. The 38.5-kDa protein is composed of three domains: D1 (M1-P60), D2 (N70-S162) and D3 (K163-S333) (Fig. 2a). D1 and D2 are connected through an interdomain linker (M61-K69). Although the corresponding linker is not well ordered in all MjTrm5b crystal structures (K68-P74), it is clearly visible in all the PaTrm5a models presented in this work. The surface of the catalytic domains D2 and D3 are rich in basic amino acids, which may contribute to the binding of tRNA (Fig. 2b). D1 consists of a four-strand β-sheet (β1–β4) and two major α-helices (α1 and α2). The sequence alignment shows poor conservation for the D1 residues among different Trm5s, while the residues in D2 and D3 domains are much more conserved (Fig. 2c). D2 comprises a six-strand β-sheet (β5–β10) and two α-helices (α3 and α4). The recognition of G37 is accomplished by β7 and β8, which form a β-hairpin. D3 corresponds to the conserved Rossmann fold, and it features a seven-strand β-sheet (β11–β17) packed against five α-helices (α6–α10).

### The recognition of substrate analogues

**Figure 3:**
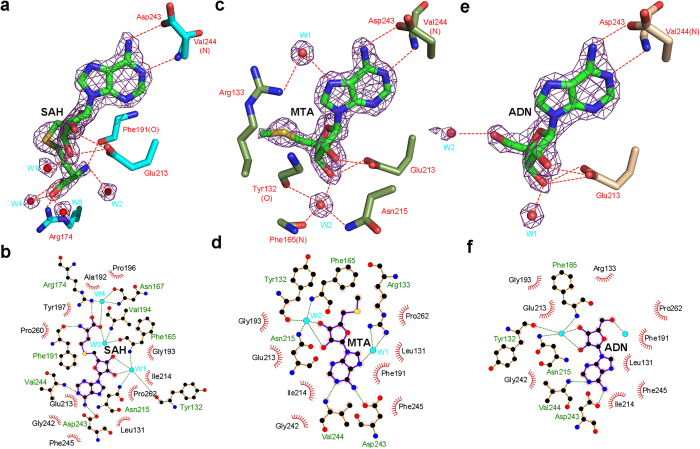
The substrate-binding modes of PaTrm5a. (**a,c,e**) The active sites of the SAH-, MTA- and ADN-bound complexes, with the ligand density of the omit map being contoured at 2σ. The residues participating in ligand recognition are depicted in sticks and labeled. The water molecules are shown as red spheres. The hydrogen bonds are shown by the red dashed lines (distance < 3.2 Å). (**b,d,f**) Interaction networks of (**a,c,e**) in cartoon representation respectively. The waters are shown as filled, light cyan balls. The hydrogen bonds are shown by dashed lines.

In the SAH-bound complex, the Ser129Gly130 dipeptide is disordered, the only disordered region in all of the four structures of PaTrm5a reported here. SAH is the product after the methyl group is abstracted from the SAM substrate. In its cocrystal structure, the adenosine moiety and the methionine group are specifically recognised by hydrogen bonding interactions with Asp243 and Val244 from D3 (Fig. 3a). The stacking interactions of the base with Phe191 and Ile214 play an additional role (Fig. 3b). The carboxyl group of the methionyl moiety hydrogen bonds to the terminal Nω atom of Arg174, while the amino group hydrogen bonds to the main chain carbonyl of Phe191. Both the 2′ and 3′ hydroxyls of the ribose form hydrogen bonds with the carboxylate oxygens of Glu213. This SAH-binding pocket also contains four well-positioned water molecules.

In addition to the product SAH, we also tried cocrystallisation of the protein with the reactive substrate SAM. Surprisingly, during the refinement process, the branched carboxylate tail of SAM could not fit the positive density in the omit map at the active site. After careful inspection of the map and subsequent refinement, we found that MTA best matches the density (Fig. 3c). This ligand was probably a degradation product of SAM before or during crystal growth (Supplementary Fig. S1d). MTA fits well in the ligand-binding pocket, and interacts with the enzyme in a way similar to that of SAH, including those involving Glu213, Asp243 and Val244 (Fig. 3a,b). Although the majority of the interactions are conserved in the complex, subtle differences are found on the orientation of Phe165. In order to engage the smaller ligand MTA, the side chain of Phe165 shifts sideways slightly, as compared to that in the SAH-PaTrm5a complex (Fig. 3b,d). Additionally, several residues around MTA may indirectly interact with the base and sugar of the ligand, via well-positioned water molecules.

To gain more insight into the mechanism of substrate recognition, we soaked or cocrystallised various nucleosides with PaTrm5a and determined the 2.2-Å crystal structure of ADN-bound PaTrm5a, which shows clear ADN density in the map. ADN is recognised by a fashion virtually identical to that of SAH or MTA: the hydrogen bonding network of the adenine ring involves a total of six residues and two well-positioned water molecules, and is further stabilised by the hydrophobic interactions with Ile214, Phe191 and the adenine ring (Fig. 3e,f).

Interestingly, the β-hairpin β7–β8 in all four structures of PaTrm5a displays large variations in B-factors (Supplementary Fig. S2a–d). The SAH-bound structure exhibits a higher degree of flexibility in this region (the Ser129Gly130 dipeptide is disordered and the side chain of Arg133 is not well defined), while the flexibility of the hairpin is relatively lower in other structures. It appears that the binding of SAH, which bears a homocysteine moiety, somehow leads to the structural flexibility of the β-hairpin. In the complex structures, the MTA-, SAH- and ADN-binding pockets align well, and the network of hydrogen bonds that stabilises SAM is maintained completely (Supplementary Fig. S2e).

### Structure comparison with MjTrm5b

**Figure 4:**
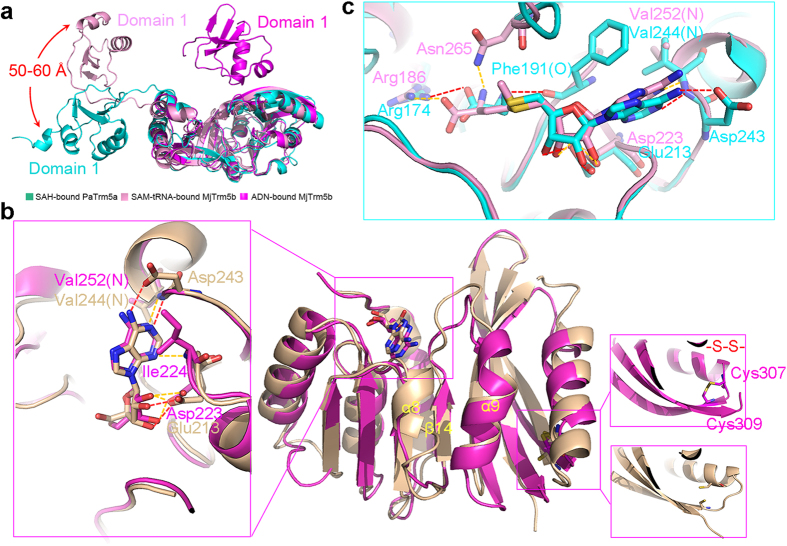
Structural comparison between PaTrm5a and MjTrm5b. (**a**) The structural superposition of the SAH-bound PaTrm5a (cyan, PDB 5HJK), ADN-bound MjTrm5b (magenta, PDB 3AY0), and SAM-tRNA-bound MjTrm5b (light violet, PDB 2ZZN). (**b**) A superposition of the ADN-bound MjTrm5b and PaTrm5a complexes. Color codes of PaTrm5 are the same as in Fig. 3e while MjTrm5b is colored magenta. Ligands are depicted in sticks. Hydrogen bonds are shown by yellow dashed lines in MjTrm5b and in red in PaTrm5a (distance < 3.2 Å). (**c**) A close-up view of the catalytic sites of the SAM-tRNA-MjTrm5b complex (PDB 2ZZN) and SAH-PaTrm5a complex structures. MjTrm5b and PaTrm5a are colored light violet and cyan respectively.

The ADN-bound MjTrm5b (PDB 3AY0, Fig. 4a, magenta) and SAM-tRNA-bound MjTrm5b (PDB 2ZZM, Fig. 4a, light violet) structures were determined at 3.05-Å and 2.65-Å resolution respectively. A pairwise superposition of the Cα atoms of the ADN-bound MjTrm5b and PaTrm5a gives an overall root-mean-square deviation (RMSD) of 3.0 Å for 211 out of the 333 atoms aligned. However, the RMSD of the D3 domains between the two structures is as low as 0.89 Å (over 100 Cα atoms). The large overall RMSD of the former may be attributed to the completely different D1 position of PaTrm5a, which adopts an extended conformation in all four structures. Compared to the ADN-bound MjTrm5b structure, D1 in PaTrm5a is displaced as far as 80 Å. In the former structure, the participating interactions involve the ENNL fragment in D1 and the KRIVK fragment in D3, including several hydrogen bonds as well as some electrostatic interactions, which are not present in PaTrm5a (Supplementary Fig. S3 and Table S1). Additionally, PaTrm5a has two longer β-strands β7 and β8 (G126-L137) in D1, and the positively charged linker between D1 and D2 is well ordered. In contrast, the corresponding fragment is mostly disordered in apo-MjTrm5b, but it becomes structured upon tRNA binding and contacts the anticodon triplet, especially G37 of tRNA^Leu^ and tRNA^Cys^ (PDBs 2ZZM and 2ZZN). According to the SAM-tRNA-MjTrm5b complex model, the free D1 domain in PaTrm5a is nowhere near the bound tRNA molecule, and it has to move 50–60 Å to make desired contacts with tRNA (Fig. 4a).

The general recognition mode of ADN is conserved between PaTrm5a and MjTrm5b with minor local structural differences. In PaTrm5a, the OD1 and OD2 atoms of Glu213 in motif 2 form hydrogen bonds with both the O2′ and O3′ hydroxyls of the ribose (Fig. 4b, left, wheat). The main chain of the Val244 forms a hydrogen bond with the N6 atom of ADN. Additionally, the SAH position in the SAH-PaTrm5a structure (Fig. 4c, cyan) is very similar to the SAM position in the ternary SAM-tRNA-MjTrm5b complex (Fig. 4c, light violet). SAH tilts moderately relative to SAM, due to the interactions with Arg174, Phe191, Glu213, Asp243 and Val244. Near the active site, the α8-helix in PaTrm5a (V244-L248) is replaced by a short loop in MjTrm5b (Fig. 4b, magenta). During the structure comparison, we found an intra-subunit disulfide bond between Cys307 from α9 and Cys309 from β16 in the MjTrm5b structure, whereas the thiol groups of the corresponding residues Cys301 and Cys308 are in the reduced form in our crystal structures (Fig. 4b). During our protein purification and crystallisation process, DTT was added to the proteins and might prevent the formation of the disulfide bond. The intra-subunit disulfide bridges between Cys301 and Cys308 may stabilise the protein at high temperatures, which may be biologically relevant to *Pyrococcus abyssi,* given its extreme growth conditions.

### Bulk FRET experiments

**Figure 5:**
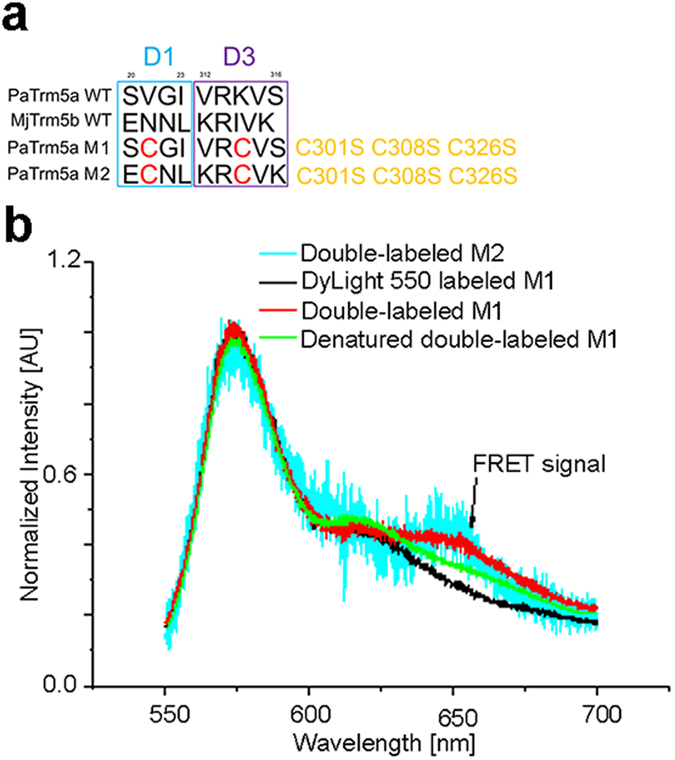
Bulk FRET experiments of PaTrm5a. (**a**) Sequences for the D1–D3 interfacial residues and the key residues for dye-conjugation after each round of mutagenesis. The M2 mutant is expected to form a closed conformation. (**b**) The bulk FRET experiment on the M1 and M2 mutants under 532-nm excitation. The horizontal axis shows the wavelength of the fluorescence light while the vertical axis shows the normalised fluorescence intensity for different samples (i.e. singly labeled M1, doubly labeled M1, doubly labeled M2 and denatured M1) excited at 532 nm and 4-μW power. The emission peaks of Dylight 550 were normalised for different samples for easy comparison of the FRET signals at the emission wavelength of 658 nm. The emission differences around 650 nm are small for both the WT and mutant samples regardless their folding states, suggesting that the most proteins are in the constitutive “open” states.

PaTrm5a displays an open conformation and our structural analyses revealed that crystal contacts are partially responsible (data not shown). To test whether in solution PaTrm5a adopts the same conformation, bulk FRET experiments were conducted. The crosslinking of the fluorophores for FRET measurements is through cysteine-coupling chemistry. First, mutagenesis in D1 and D3 was performed on PaTrm5a to remove unwanted cysteine residues to minimise the interference with covalent labeling of the fluorophores. There are only three cysteine residues in PaTrm5a, present at positions 301, 308 and 326 respectively. These undesired cysteines (judged from their positions in the structure) are all located in D3 and they were mutated to serines. Next, we performed another round of mutagenesis, which allowed us to chemically couple the fluorescence dyes. According to our structures, Val21 from D1 and Lys314 from D3 were both mutated to cysteines to give the mutant M1 in order to observe the D1–D3 interdomain movements by FRET. Then we generated the mutant M2 with the sequence ECNL at positions 20 to 23 and KRCVK at positions 312–316. The purpose of the M2 mutations is to re-create the contacts between the D1 and D3 domains of MjTrm5b in PaTrm5a. We expect that these mutations would tether the two domains together, and M2 thus forms a “closed” conformation (Fig. 5a). Therefore, the M1 mutant mimics the WT PaTrm5a enzyme, whereas the M2 mutant mimics MjTrm5b (Supplementary Fig. S3, the left panel, and Table S1). The close distance between D1 and D3 in M2 would produce strong energy transfer between the donor and acceptor, if a closed conformation can be engineered through the aforementioned mutations.

We then performed the FRET experiments to examine the changes in the relative distance between D1 and D3 in solution conditions. The Dylight 550 and 633 fluorescent dyes were chosen as the FRET pair because of the small overlap between the excitation peak of donor (Dylight 550) and the emission peak of the acceptor (Dylight 633) (Supplementary Fig. S4). Previously, dyes with similar fluorescent properties have been used as an efficient FRET pair to measure the oligomerization efficiency of the protein IFI16 in the presence of excess DNA^20^. Assuming equal labeling efficiency at each site, the two cysteines (Cys21 and Cys314) will be evenly labeled, and the total amount of dyes (including both Dylight 550 or 633) present in the doubly labeled proteins will be the same as their singly labeled counterparts. The labeled proteins were subjected to fractionation by a heparin column and gel analyses, and fractions with slightly higher molecular-mass were used for the FRET experiments due to the size addition of the dyes (Supplementary Fig. S5). We also checked the integrity of the M1 and M2 mutants by gel-filtration chromatography analyses. All proteins showed similar chromatograms, suggesting that the mutations and dye-conjugation did not significantly perturb their local structures (data not shown).

As a confirmation of successful labeling, we measured the emission spectra of the labeled PaTrm5a, which gave out strong fluorescence emission under the corresponding excitation wavelength at 532 nm, or 632 nm (Fig. 5b) respectively, suggesting excellent labeling efficiencies of both dyes. However, as shown in Fig. 5b, the doubly labeled M1 or M2 mutant, when excited at 532 nm, only gave a very small amount of fluorescence at 658 nm (the wavelength of the emission peak of the acceptor dye). The FRET efficiency is thereby quite low, supporting the conclusion that, for the majority of the PaTrm5a molecules, the protein is in an “open” state. This result is consistent with the observation in the crystal structures. As a negative control, we also measured the FRET intensity of completely denatured doubly labeled Trm5a by urea (heat denaturation was avoided due to the high thermostability of PaTrm5a). The fluorescence at 658 nm for denatured Trm5a was only slightly smaller than that of the M1 or M2 mutants in the native state, indicating that, even in solution, PaTrm5a adopts the open conformation as observed in the crystal structures.

## Discussion

In this study, we carried out structural studies on the bifunctional methyltransferase PaTrm5a. Of the four high-resolution structures we obtained, we unexpectedly cocrystallised an MTA-PaTrm5a complex. In retrospect, the commercially available SAM was 80–90% pure, and was probably degraded on repeated freeze-and-thaw processes. We later tested its stability and discovered that ~10% SAM was degraded per day at 25 °C, when preserved in a buffer containing 20 mM HEPES (pH 7.0). The degradation product MTA can be separated from SAM by TLC and detected by UV light (Supplementary Fig. S6). Lahoud *et al.* tested SAM derivatives for their competitive inhibition against MjTrm5b^18^ and found that the *K*_i_ value of MTA for MjTrm5b is only higher than the *K*_m_ of SAM by 2.8-fold. With similar catalytic domains (D2 and D3), it is possible for PaTrm5a to cocrys-tallise with MTA under the crystallisation conditions.

The four structures of PaTrm5a reported in this study all adopt an “open” or extended conformation distinct from that of Trm5b. To further characterize the “unfolded” state in PaTrm5a, we performed FRET experiments to study the distance changes between D1 and D3 in solution. Structure-based design in M2 to engineer an interface similar to that between D1 and D3 present in MjTrm5b was largely unsuccessful, suggesting greater flexibility of D1 in PaTrm5a, although M2 only had one of the two contacting regions from MjTrm5b. The results suggested that the protein is constantly “unfolded” as observed in the crystalline state, and therefore the open conformation in the crystal structures is a genuine state of this unique enzyme. SAXS studies of PaTrm5a are currently underway to verify our FRET results.

The D1 domain is a small conserved domain and features two helices packed against a four-strand sheet. Its evolutionary origin is not clear from a structural perspective, because a Dali search for structural homologues using the D1 structure did not return plausible hits. However, overlay of the D1 domain from Patrm5a with that of MjTrm5b produced a good alignment of Cα backbones (RMSD of 1.9 Å for 59 Cα atoms aligned, Supplementary Fig. S7), although their D1 domains only share ~30% sequence identity. Relatively large structural differences occur in α2 and the following loop. In MjTrm5b, two fragments (Glu21–Asn2323 and Asp46–Asp48) interact with residues in the D3 domain to produce the closed conformation. These residues are mostly non-conserved in D1 of PaTrm5a, and thus lead to its open conformation (Supplementary Fig. S8). Blast search shows that the D1 domain of PaTrm5a is a sequence-conserved domain in many thermophilic archaea, and it share 40–60% sequence identities with these closely related Trm5s (Supplementary Fig. S8). Multiple sequence alignment demonstrated that the corresponding residues are not conserved in these homologues (presumably aTrm5as) either, suggesting that they also adopt extended structures. On the other hand, D1 is a tRNA-recognition domain. In fact, it is named as the “sensor domain”, responsible for recognising the outer corner of tRNA^19^. Of note, with the exception of Asn23, none of the D1 residues in MjTrm5b responsible for tRNA interactions overlaps with those responsible for the D3-interactions. As shown by Supplementary Fig. S8, the tRNA-contacting residues are not strictly conserved either in PaTrm5a or its homologues. We further aligned members of the Trm5a, Trm5b, and Trm5c subfamilies, and discovered that D1 is missing in 1/3-analysed Trm5as (data not shown). Therefore, the tRNA-recognition mechanism employed by Trm5as may be different from those by Trm5bs and Trm5cs. Furthermore, the extended conformation that PaTrm5a exhibits is also dissimilar to the “open” conformation of tRNA-bound MjTrm5b (Fig. 4a). The current conformation of PaTrm5a has higher entropy and Gibbs energy (ΔG) than the well-folded state, implying that the kinetic behaviors during the enzymatic reactions are different. Taken together, the D1 domain is an interesting domain that contacts both the D3 domain and tRNA substrates in Trm5bs and Trm5cs. We infer that during the evolutionary process, it lost the ability to interact with D3 and only retained the ability to bind tRNA in aTrm5as. The consistency of the extended conformation in all four structures may have functional implications for the bifunctional transferase, which deserves further investigation.

## Methods

### Cloning, expression and purification of PaTrm5a

The wild type (WT), full-length PaTrm5a gene was chemically synthesized (Genewiz, Suzhou, China) and PCR-amplified. After the double digestion by the restriction enzymes NdeI and XhoI, the digested PCR product was inserted into a modified pET-28a (+) vector, where the thrombin recognition site (LVPRGS) in pET-28a (+) was replaced by the PreScission protease cleavage site LEVLFQGP. For the FRET experiments, several rounds of PaTrm5a mutagenesis were carried out for fluorophore-labeling: in the first round, the unwanted cysteines in WT PaTrm5a were mutated using three consecutive *QuikChange* (Stratagene) PCRs to produce the triple mutant C301S/C308S/C326S with the *QuikChange* primers (including the sense and antisense primers) for the C301S, C308S and C326S mutations respectively (Supplementary Table S2); in the second round, two cysteines were introduced into the triple mutant using two more *QuikChange* PCRs with the primers for the V21C and K314C mutations to produce the mutant M1 (with the mutations V21C/C301S/C308S/K314C/C326S); in the last round, the mutant M2 was generated from M1 using two PCRs through the SCGI-to-ECNL and VRCVS-to-KRCVK primers. All the amplifying and *QuikChange* primers used in this study were listed in Supplementary Table S2.

The plasmids of PaTrm5a and mutants were transformed into the *Escherichia coli* strain BL21 (DE3) cells for overexpression. The harvested cells were lysed by sonication. The cell debris was removed by centrifugation, and the supernatant was then applied onto a Ni-NTA affinity column (Qiagen). In order to remove the endogenous nucleic acid bound to the recombinant PaTrm5a, the eluted fractions were collected and treated overnight with 0.08 mg/mL RNase A in the presence of 5 mM MgCl_2_. The protein was further subjected to affinity purification by a heparin column (GE Healthcare) using a NaCl gradient, and PaTrm5a was eluted at ~500 mM NaCl. The purified protein was pooled, dialysed in a buffer containing 20 mM Tris-HCl (pH 8.0), 150 mM NaCl and 1 mM DTT (without DTT for the M1 mutant) and stored after being flash-frozen.

### Crystallisation

The purified WT PaTrm5a was concentrated to 20 mg mL^−1^. Crystallisation samples with SAH or SAM were prepared by mixing PaTrm5a with 1.5 mM SAH or SAM at a protein: ligand molar ratio of 1:3. Crystallisation was performed using the sitting-drop vapor diffusion method at 25 °C, and the sample and the well solution were mixed at a 1:1 volume ratio. Microcrystals of PaTrm5a appeared in a condition containing 10–15% (w/v) PEG 3350, 50 mM HEPES (pH 7.5), 150 mM Mg(OAc)_2_ and 50 mM KCl during the initial screens. After optimisation, high-quality crystals were obtained in 12% (w/v) PEG 3350, 100 mM HEPES (pH 7.5), 100 mM Ca(OAc)_2_ and 100 mM KCl.

In order to obtain high-quality crystals for data collection, the iterative seeding method was employed. Microseed solutions were prepared as follows: 5–15 small crystals were harvested, washed, diluted with reservoir liquor and transferred to an eppendorf tube, where they were crushed with a seed bead. Then the resuspended solution was used and was added to the well-equilibrated crystallisation drops. Once new crystals appeared, these crystals will be chosen for the next round of seeding.

The fully-grown crystals were soaked in a cryoprotectant solution containing all the components of the reservoir solution supplemented by 20% ethylene glycol (v/v). The soaked crystals were mounted on nylon loops and flash frozen in liquid nitrogen.

### Data collection and structure determination

Native data of PaTrm5a bound with SAH and SAM were collected at −173 °C at the BL19U1 and BL17U1 beamlines in Shanghai Synchrotron Radiation Facility (SSRF, Shanghai, P.R. China) respectively, and the data were processed with the program *HKL2000*^21^. The native data of the apo-protein and the ADN-cocrystals were collected using an Oxford Diffraction Xcalibur Nova diffractometer. The diffractometer was operated at 50 kV and 0.8 mA, with a rotation of 1° per frame at −120 °C. The data were recorded using a 65 mm Onyx CCD detector, and the exposure time was 90 s for each frame. The complete dataset was processed using CrysAlisPro (v.1.171.33.49; Oxford Diffraction) and scaled using *SCALA* from the *CCP4* suite^22^.

To solve the structure of the SAH-bound cocrystals, molecular replacement (MR) was first performed using *PHENIX*^23^ with the coordinates the MjTrm5b structure (PDB code 3AY0)^18^ as the search model. Cell content analysis indicated that there was one molecule in the asymmetric unit. A partial solution was found with only the D3 domain (residues P168-S333) matching the electron density after the molecular replacement run. To obtain the complete protein structure, several rounds of density modification were carried out to obtain an interpretable map for the entire protein. The rest part of the protein was further built manually according to the electron density map with *COOT* 24. The resulting structure of PaTrm5a displays a distinctive conformation from that of MjTrm5b. Multiple cycles of refinement alternating with model rebuilding was carried out by *PHENIX.refine*^23^, and the final model was validated by *SFCHECK* 25. This structure was in turn used for the search model for the other structures of PaTrm5a reported in this study. The structural figures were produced with *PyMOL* (http://www.pymol.org/). All data collection and refinement statistics are presented in Table 1. The cartoons representing the interaction network as shown in Fig. 3b,d, f were generated by *LIGPLOT *26.

### Fluorescence resonance energy transfer

The purified M1 and M2 mutants were present in a buffer containing 20 mM Tris-HCl at pH 8.0, 150 mM NaCl and 1 mM DTT. Before labeling, the protein was reduced with 10 mM DTT for 2 hours at 4 °C, which broke the disulfide bonds thoroughly. The removal of the reducing reagent from the protein samples was performed by dialysis against 1xPBS (pH 7.3) for 4 hours at 4 °C. To doubly label the protein with the acceptor and donor dyes, 0.75-fold (in molar ratio) of Dylight 633 maleimide (Thermo, USA) and 0.75-fold of Dylight 550 maleimide (Thermo, USA) were added to the protein solution simultaneously, and the mixture was incubated at 4 °C for overnight. For single labeling, 1.50-fold donor or acceptor dye was added and the same protocol was followed. The labeled protein was isolated from the unreacted dye by a heparin column (GE Healthcare) in a HEPES buffer (20 mM HEPES, pH 7.0) with increasing NaCl concentration to 1 M. Fractions of each peak were collected for gel analyses, and we chose the protein fractions with a slightly higher molecular-mass because the dyes are ~1 kDa in size. The target protein was then dialysed into a buffer containing 20 mM HEPES (pH 7.0) and 150 mM NaCl. To prepare completely denatured PaTrm5a, we incubated 7 M urea with the doubly labeled WT protein for 4 hours for complete denaturation of the protein. All the protein samples were filtered by a membrane filter with 0.22-μm pores (Merck Millipore) before the FRET measurements. The bulk fluorescence spectra were obtained using a fluorescence spectrometer (F-2500, Hitachi High Technologies, Tokyo, Japan) at the room temperature. Typically, a 40-μL sample with 8 μM protein was injected into the sample cell. The excitation light at 532 nm was produced from a solid-state laser (SDL-532-LN-150T, Shanghai Dream Lasers Technology, Shanghai, China). The fluorescence passed through the dichroic mirror (Di01-R550-25 × 36, Semrock, Rochester, NY) and was spatially filtered by a slit set parallel to the excitation spot. The excitation light at 632 nm was produced from a solid-state laser (SDL-632-LN-150T, Shanghai Dream Lasers Technology, Shanghai, China). The fluorescence passed through the dichroic mirror (Di01-R630-25 × 36, Semrock, Rochester, NY). The exposure time was 5 s and the excitation irradiance was 4 μW.

### Thin-layer & chromatography

The SAM aliquots in 20 mM HEPES (pH 7.0) were taken and spotted on a cellulose polyethyleneimine TLC plate (Merck Millipore). The spots were 2 cm from the bottom of the plate and 1 cm apart, and the plates were developed with *n*-BuOH-HOAc-H_2_O (12:3:5) as the solvents in a glass jar. Then the TLC plates were exposed to UV light for visualization.

## Additional Information

**Accession codes:** Atomic coordinates and structure factors have been deposited in the Protein Data Bank under accession numbers 5HJI, 5HJJ, 5HJK and 5HJM for ADN-PaTrm5a, apo-PaTrm5a, SAH- PaTrm5a and MTA-PaTrm5a structures.

**How to cite this article**: Wang, C. *et al.* Crystal structures of the bifunctional tRNA methyltransferase Trm5a. *Sci. Rep.* **6**, 33553; doi: 10.1038/srep33553 (2016).

## Supplementary Material

Supplementary Information

## Figures and Tables

**Figure 1:**
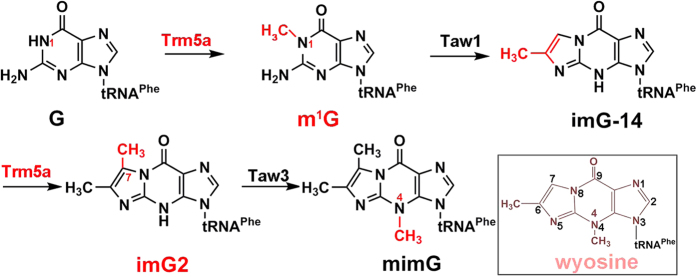
The biosynthetic pathway of mimG in *Pyrococcus abyssi*. The specific additions by each enzyme are highlighted in red. The chemical structure of the wyosine is drawn in the box and the numbering is also shown.

**Table 1:** Data collection and refinement statistics.

	Apo-PaTrm5a (5HJJ)	PaTrm5a-SAH (5HJK)	PaTrm5a-MTA (5HJM)	PaTrm5a-ADN (5HJI)
**Data collection**				
Beamlines	In-house X-ray source	BL19U1	BL17U1	In-house X-ray source
Resolution range (Å)	25.80–2.00 (2.11–2.00)^a^	50.00–2.00 (2.07–2.00)	50.00–1.76 (1.82–1.76)	24.57–2.20 (2.32–2.20)
Space group	*P* 2_1_2_1_2_1_	*P* 2_1_2_1_2_1_	*P* 2_1_2_1_2_1_	*P* 2_1_2_1_2_1_
Cell dimensions (Å)				
*a, b, c* (Å)	52.83, 54.32, 129.02	52.85, 54.52, 129.97	52.97, 55.33, 130.38	52.73, 54.72, 128.95
*α, β, γ* (°)	90, 90, 90	90, 90, 90	90, 90, 90	90, 90, 90
R_merge_	0.14 (0.74)	0.15 (0.38)	0.07 (0.32)	0.13 (0.55)
Redundancy	3.8 (3.5)	12.5 (11.5)	7.0 (7.1)	3.4 (3.2)
Completeness (%)	99.5 (94.1)	100 (100)	99.4 (95.6)	99.2 (91.3)
*I/σ*_*(I)*_	7.9 (1.7)	18.79 (5.29)	25.07 (6.51)	8.0 (2.3)
**Refinement**				
Resolution range (Å)	24.56–2.00 (2.08–2.00)	26.43–2.00 (2.07–2.00)	36.72–1.76 (1.81–1.76)	24.57–2.20 (2.31–2.20)
No. reflections	25661	26146	38622	19348
R_work_/R_free_^b^(%)	19.7/24.5	17.7/21.7	17.6/20.3	19.3/23.6
No. atoms				
Protein	2805	2811	2841	2830
Ligand	—	26 (SAH)	20 (MTA)	19 (ADN)
Water	332	257	408	243
B-factor (Å^2^)				
Protein	25.01	31.15	24.81	26.24
Ligand	—	24.76 (SAH)	15.09 (MTA)/36.12 (Rest)	19.97 (ADN)
Water	31.60	37.49	35.24	29.20
RMS (bonds) (Å)	0.004	0.007	0.004	0.003
RMS (angles) (°)	0.89	1.19	0.89	0.81
Ramachandran favored (%)	97.98	99.13	99.14	98.56
Outliers (%)	0.00	0.00	0.00	0.29

^a^Values in parentheses are for the highest-resolution shell.

^b^The free R-factor was calculated using 5% of reflections omitted from the refinement.
