# Supplementary material for: Crystal structures of the bifunctional tRNA methyltransferase Trm5a
Source: Sci Rep. 2016 Sep 15;6:33553. doi: 10.1038/srep33553 (PMC5024318; doi:10.1038/srep33553)
Supplement: Supplementary Information [file srep33553-s1.doc]

Supplementary Information for

**Crystal structures of the bifunctional tRNA methyltransferase Trm5a**

Caiyan Wang^1,2, §^, Qian Jia^1,2, §^, Ran Chen^1,2^, Yuming Wei^3^, Juntao Li^3^, Jie Ma^3^, Wei Xie^1,2^,*

^1^State Key Laboratory for Biocontrol, School of Life Sciences, The Sun Yat-Sen University, Guangzhou, Guangdong 510275, People's Republic of China

^2^Center for Cellular & Structural biology, The Sun Yat-Sen University, 132 E. Circle Rd., University City, Guangzhou, Guangdong 510006, People's Republic of China

^3^State Key Laboratory of Optoelectronic Materials & Technologies, School of Physics, Sun Yat-Sen University, Guangzhou, Guangdong 510006, China

^§^These authors contributed equally to this work.

*To whom correspondence should be addressed.

Tel.: 862039332943; Fax: 862039332847; Email: [xiewei6@mail.sysu.edu.cn](mailto:xiewei6@mail.sysu.edu.cn).

**Contents**

Supplementary Figures S1-8 and Figure Legends

Supplementary Tables S1-2

**
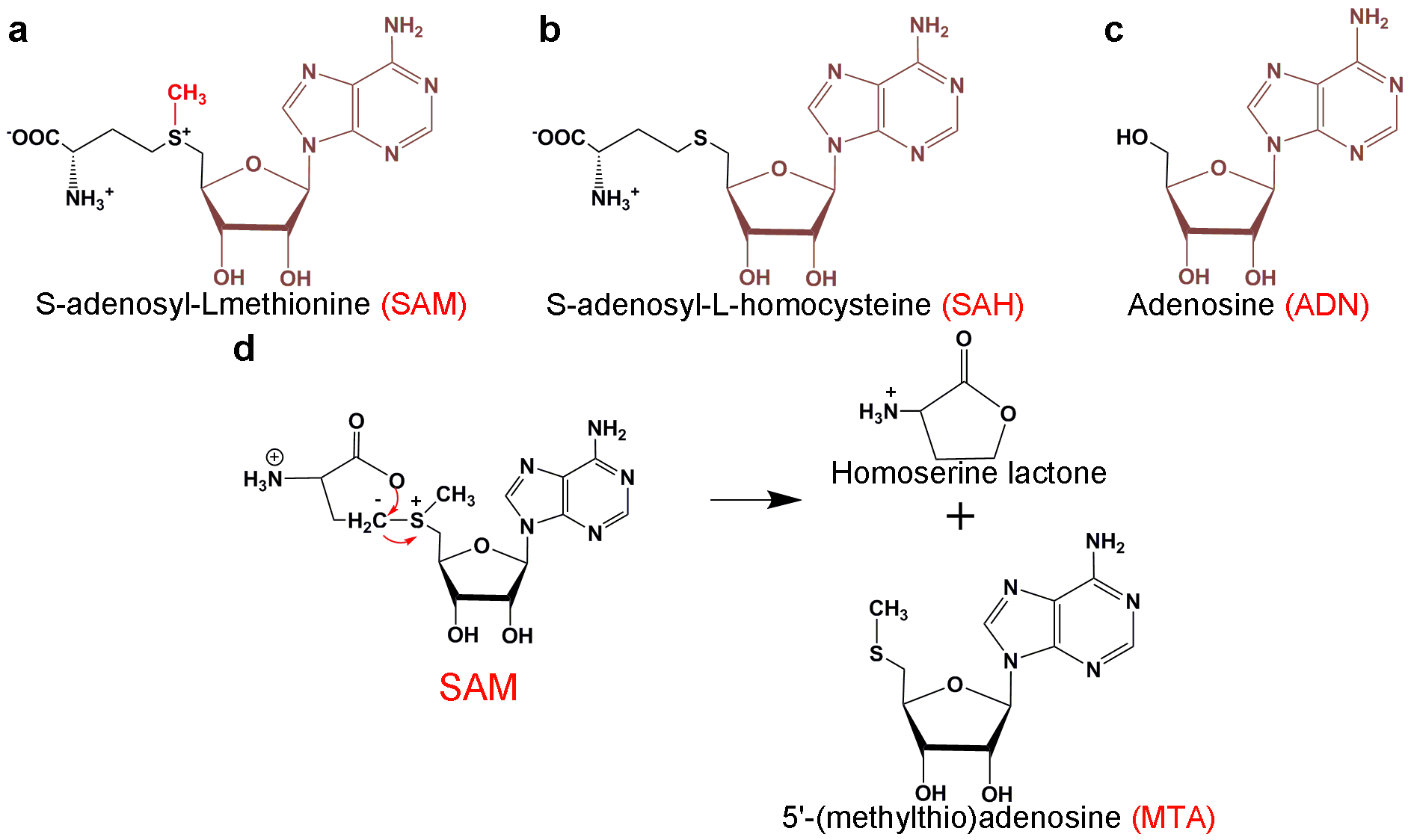
**

**Supplementary Figure S1. The chemical structures of the** **ligands used in this study.** (a) SAM, (b) SAH, and (c) ADN. (d) A schematic representation of the degradation mechanism of SAM to produce MTA.


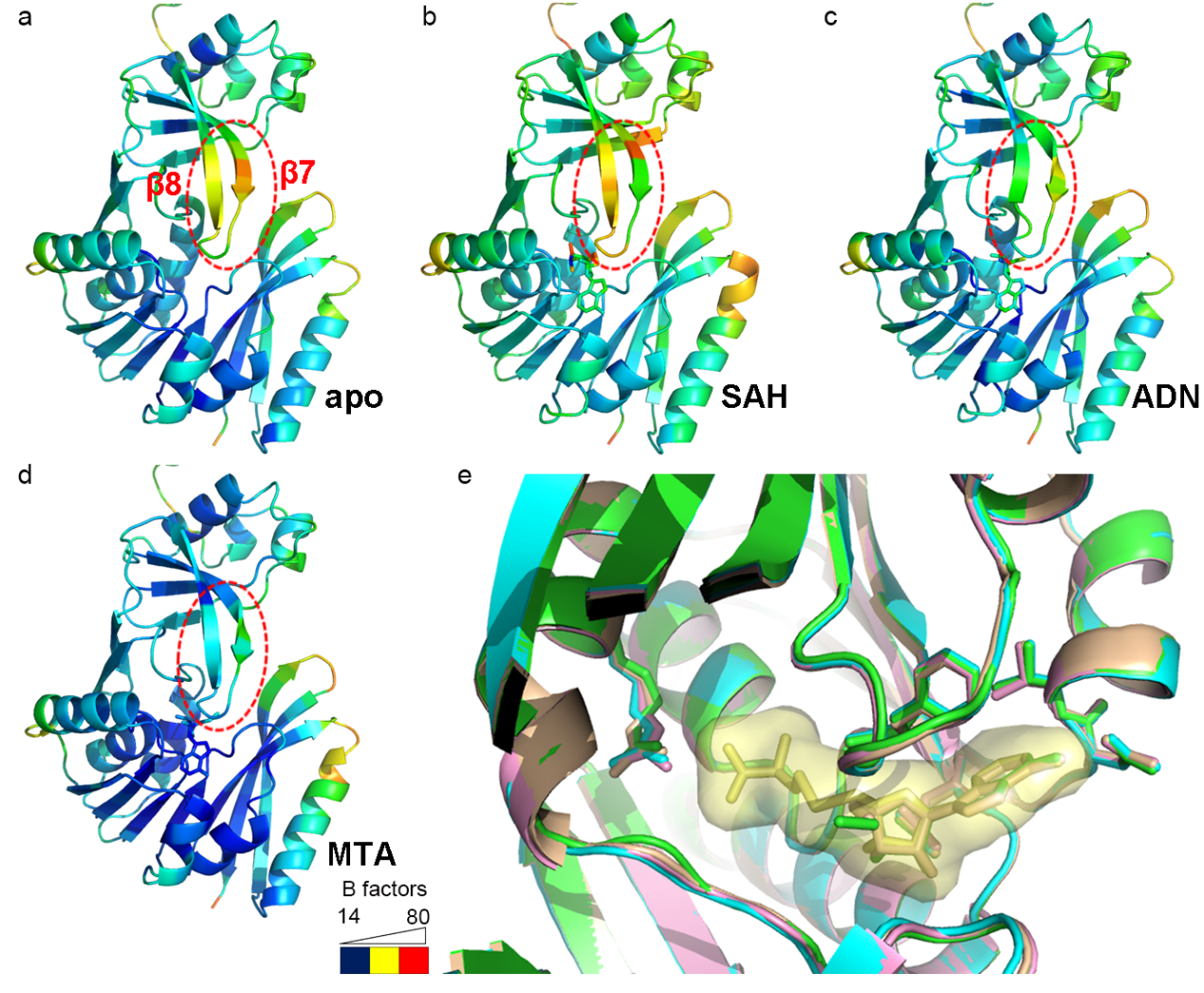


**Supplementary Figure S2.** The structural changes induced by interactions with the substrate or analogs. (a-d) The structures of the apo, SAH, ADN and MTA-PaTrm5a are colored according to their B-factor spectra, with cold colors representing the more stable regions while hot colors representing the more flexible regions. (e) The structure superposition of the SAH-, MTA- and ADN-bound complexes colored in cyan, green and violet respectively. The residues for the recognition are shown by the ball-and-stick model. The β7-β8 hairpin is circled and labeled.


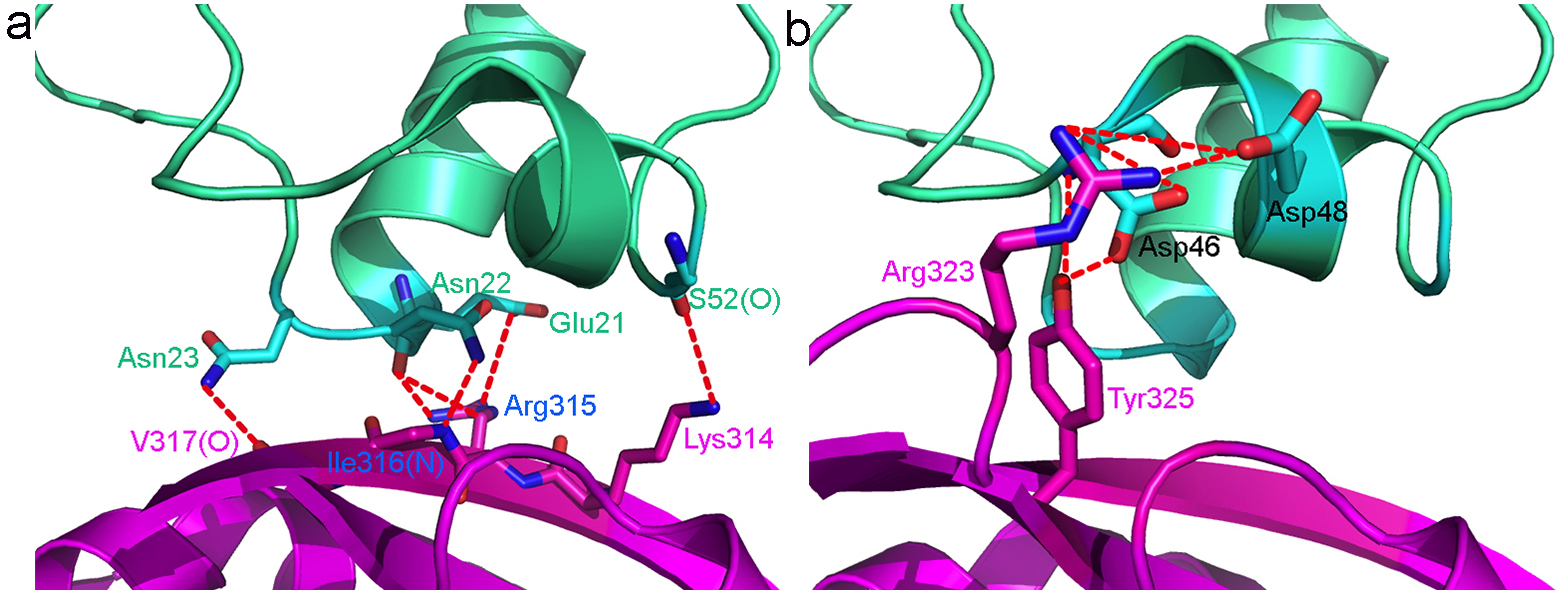


**Supplementary Figure S**3. The detailed interaction pattern between D1 (cyan) and D3 (magenta). Hydrogen bonds and salt bridges are shown as red dashed lines.


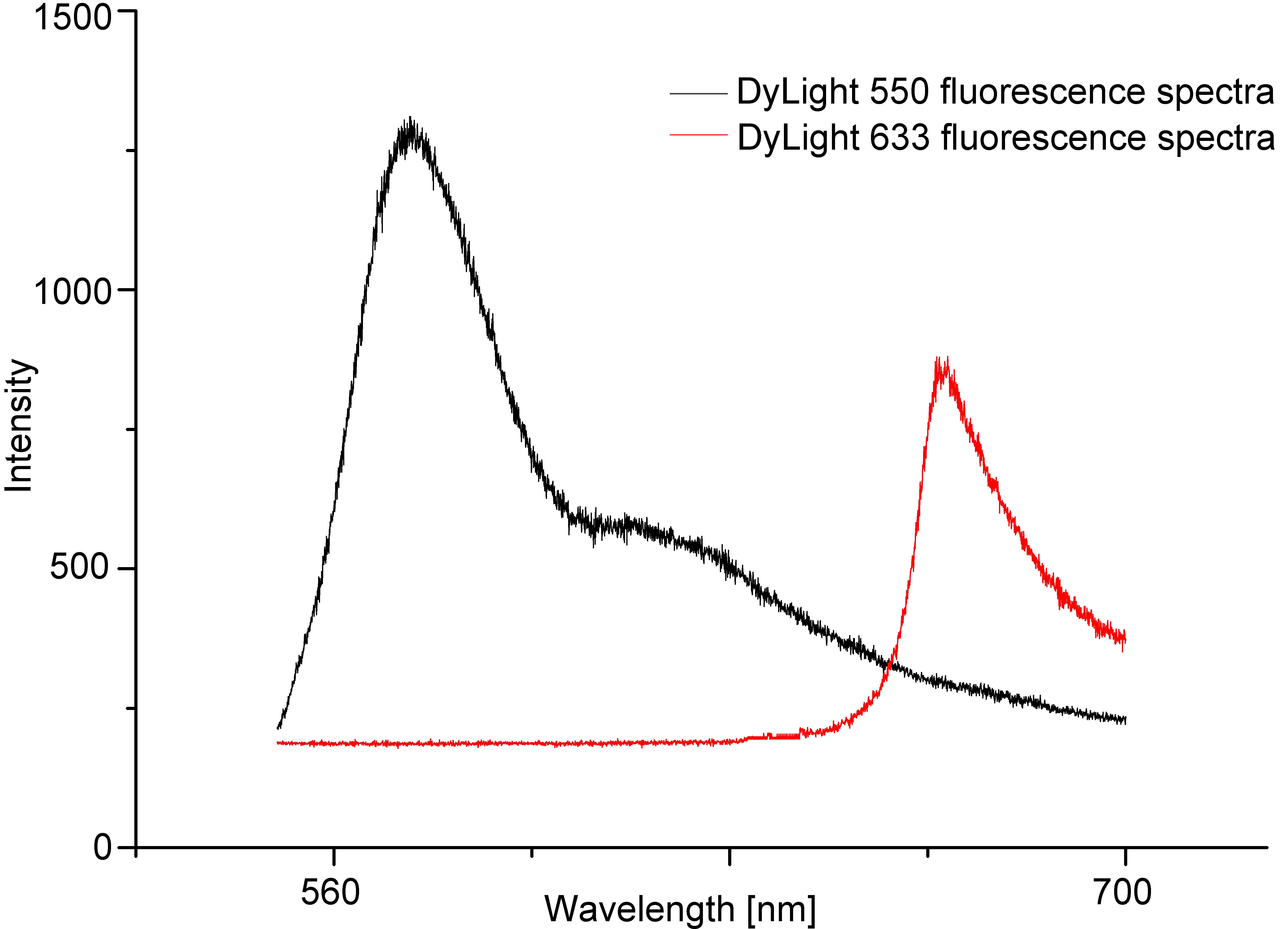


**Supplementary Figure S4.** The fluorescence spectra of DyLight 550 and DyLight 633 emission. The horizontal axis shows the wavelength of the fluorescence light while the vertical axis shows the fluorescence intensity. Dylight 550 was excited at 532 nm and with 4-μW power while the Dylight 633 was excited at 632 nm and with 4-μW power.


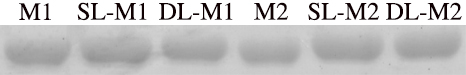


**Supplementary Figure S5.** The SDS-PAGE gel analyses of the protein samples after fluorophore-labeling. Ctrl, the unlabeled WT or mutant; SL, singly labeled WT by Dylight 550 or 633; DL, doubly labeled mutant by Dylight 550 or 633.


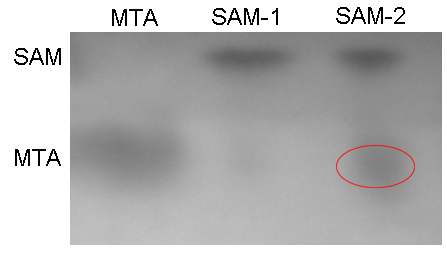


**Supplementary Figure S6.** SAM quality check by TLC chromatography. MTA: the commercially purchased 5’-methylthioadenosine; SAM-1: fresh-made SAM sample by dissolving the commercial SAM powder in a buffer containing 20 mM HEPES (pH 7.0); SAM-2: the same sample left at 25 °C overnight. The migration positions of SAM and the degradation product MTA are indicated on the left.


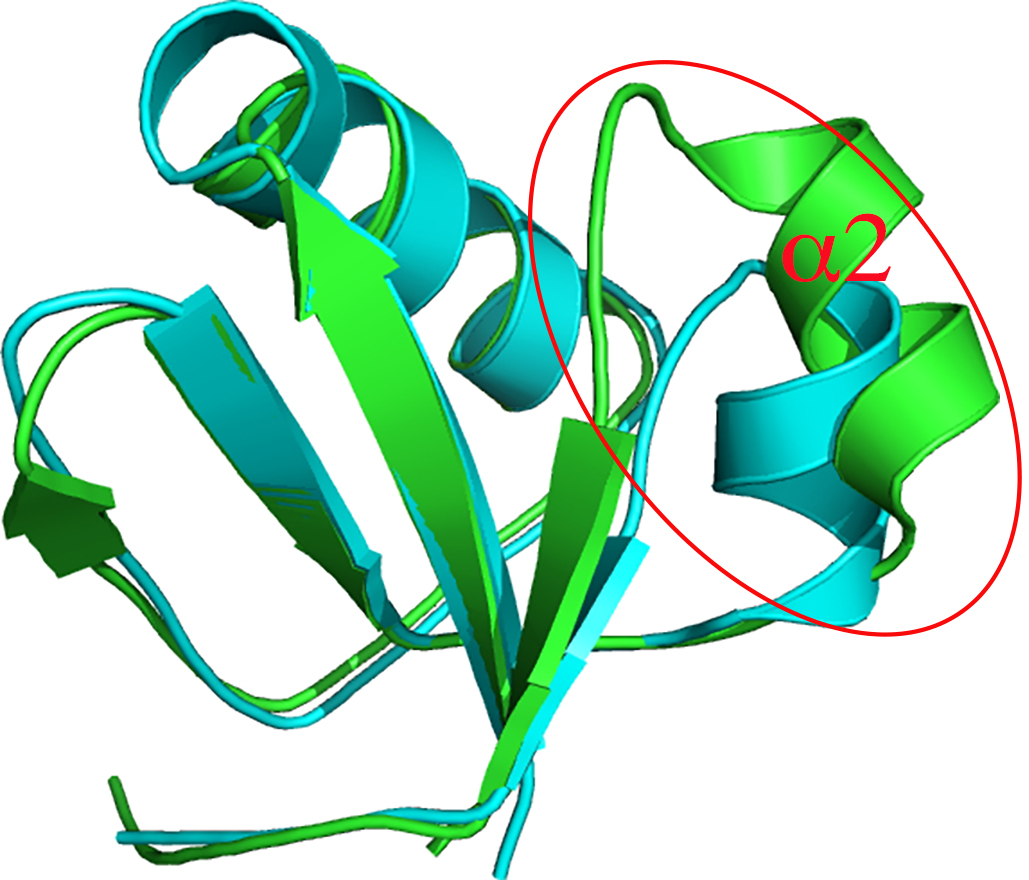


**Supplementary Figure S7.** Overlay of the D1 domain of PaTrm5a with MjTrm5b. The region with large structural differences is circled.

**
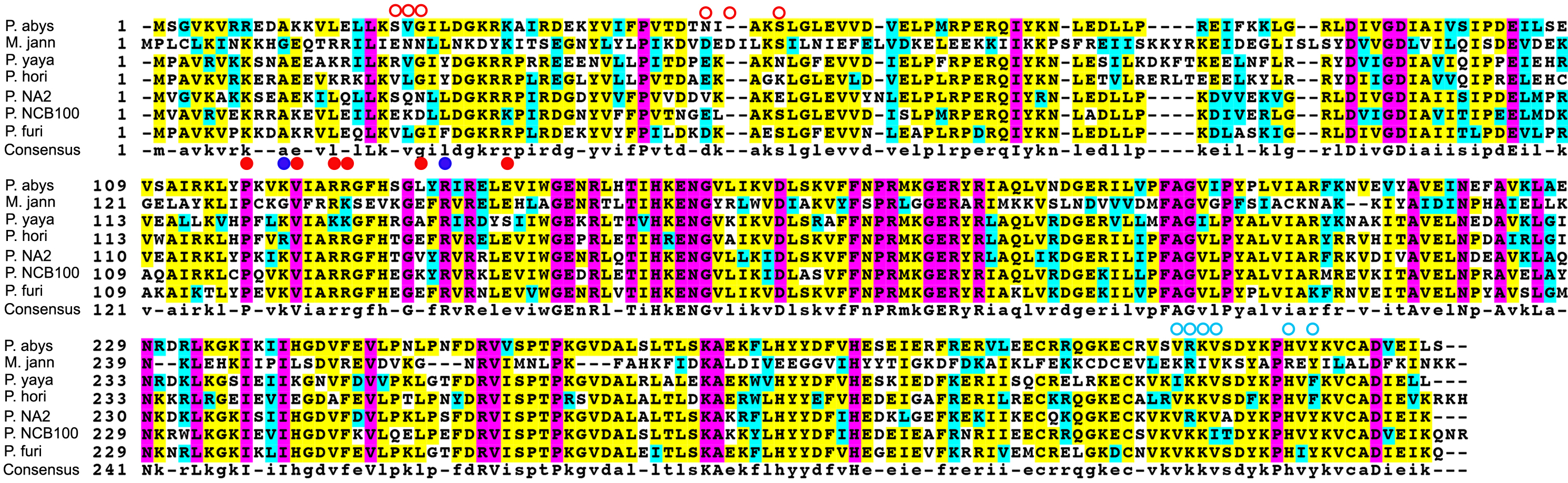
**

**Supplementary Figure S8.** Multiple sequence alignment of the D1 domain of PaTrm5a from the Blast search. Listed sequences are PaTrm5a (P. abys), MjTrm5b (M. jann), putative Trm5s from *Pyrococcus yayanosii* (P. yaya), *Pyrococcus horikoshii* (P. hori), *Pyrococcus sp. NA2* (P. NA2), *Pyrococcus sp. NCB100* (P. NCB100), and *Pyrococcus furiosus* (P. furi). Identical residues in sequences are in magenta and yellow, and similar residues in cyan. The important residues for D1-D3 contacts in MjTrm5b are indicated by open circles on the top, while the important residues in D1 for tRNA contacts are indicated by solid circles at the bottom. Open and cyan red circles designate residues from the D1 and D3 domains respectively; red and blue solid circles represent contacts via side chains and main chains respectively.

**Supplementary Table S1**. Specific interactions between the D1 domain and the D3 domain of MjTrm5b (PDB 2YX1). Hydrogen bond distance less than 4.0 Å, and salt bridges less than 4.5 Å are listed.

| Domain 1 | | Domain 3 | | Distance  (angstroms) |
| --- | --- | --- | --- | --- |
| Residue | Atom | Residue | Atom |
| E(Glu)21 | O | R(Arg)315 | NH1 | 2.99 |
| E(Glu)21 | O | R(Arg)315 | NE | 3.91 |
| E(Glu)21 | OE1 | R(Arg)315 | NH2 | 3.85 |
| N(Asn)22 | ND2 | I(Ile)316 | N | 2.90 |
| N(Asn)23 | ND2 | V(Val)317 | O | 2.89 |
| S(Ser)52 | O | K(Lys) 314 | NZ | 3.34 |
| D(Asp)46 | N | Y(Tyr)325 | OH | 3.03 |
| D(Asp)46 | OD2 | Y(Tyr)325 | OH | 2.61 |
| D(Asp)46 | OD1 | R(Arg)323 | NH2 | 3.78 |
| D(Asp)48 | OD2 | R(Arg)323 | NH2 | 2.39 |
| D(Asp)48 | OD1 | R(Arg)323 | NH2 | 4.47 |
| D(Asp)48 | OD2 | R(Arg)323 | NH1 | 4.34 |

**Supplementary Table S2.** The primers used in this study. The mutated bases in the *Quikchange* primers are underlined.

| Gene | Sense Primers | Antisense Primers |
| --- | --- | --- |
| WT | 5’-GCGGCAGCCATATGAGTGGCGTGAAAGTTCGC-3’ | 5’-TTGCACTTCTCGAGTCAACTCAGAATCTCCACGT-3’ |
| V21C | 5’-GGAGCTGCTGAAAAGCTGCGGCATCCTGGATGG-3’ | 5’-CCATCCAGGATGCCCGAGCTTTTCAGCAGCTCC-3’ |
| C301S | 5’-CGTTCTGGAAGAAAGCCGCCGCCAGGG-3’ | 5’-CCCTGGCGGCGGCTTTCTTCCAGAACG-3’ |
| C308S | 5’-CCGCCAGGGTAAAGAAAGCCGTGTGAGTAAGCG-3’ | 5’-CGCTTACTCACACGGCTTTCTTTACCCTGGCGG-3’ |
| K314C | 5’-CCGTGTGAGTGTTCGCTGTGTGAGCGATTACAAAC-3’ | 5’-GTTTGTAATCGCTCACACAGCGAACACTCACACGG-3’ |
| C326S | 5’-GTGTATAAAGTGAGTGCCGACGTGG-3’ | 5’-CCACGTCGGCACTCACTTTATACAC-3’ |
| SCGI-to-ECNL | 5’-GAGTGCAATCTCCTGGATGGTAAACGCAAGGCCATCCGT-3’ | 5’-TTTCAGCAGCTCCAGCACTTTCTTTGC-3’ |
| VRCVS-to-KRCVK | 5’-AAGCGATGCGTAAAGGATTACAAACCGCACGTGTATAAA-3’ | 5’-ACTCACACGGCATTCTTTACCCTG-3’ |
